# HPLC estimation of iothalamate to measure glomerular filtration rate in humans

**DOI:** 10.1186/s13065-016-0227-3

**Published:** 2016-12-12

**Authors:** Iltaf Shah, James Barker, Declan P. Naughton, Stephen J. Barton, Syed Salman Ashraf

**Affiliations:** 1 School of Life Sciences, Pharmacy and Chemistry, Kingston University, Penrhyn Road, Kingston-upon-Thames, Surrey, KT1 2EE UK; 2 Department of Chemistry, College of Science, United Arab Emirates University, Al Ain, UAE

**Keywords:** Iothalamate, Iothalamic acid, GFR, Glomerular filtration rate, Human urine and plasma, HPLC

## Abstract

Glomerular filtration rate (GFR) is usually determined by estimation of iothalamate (IOT) clearance. We have developed and validated an accurate and robust method for the analysis of IOT in human plasma and urine. The mobile phase consisted of methanol and 50 mM sodium phosphate (10:90; v/v). Flow rate was 1.2 mL/min on a C18 reverse phase column, Synergi-hydro (250 × 4.6 mm) 4 µm 80 Å, with an ultraviolet detector set to 254 nm. Acetonitrile was used for the deproteination and extraction of IOT from human plasma and urine. Precision and accuracy were within 15% for IOT in both plasma and urine. The recoveries of IOT in urine and plasma ranged between 93.14% and 114.74 and 96.04–118.38%, respectively. The linear range for urine and plasma assays were 25–1500 and 1–150 µg/mL respectively. The lower limits of detection were 0.5 µg/mL for both urine and plasma, with no interference from plasma and urine matices. This method has been fully validated according to FDA guidelines and the new HPLC assay has been applied to a new formulation of IOT (Conray™ 43), to calculate GFR in healthy volunteers. The new method is simple, less expensive and it would be instrumental in future clinical and pharmacokinetic studies of iothalamate in kidney patients.

## Background

Renal function is best studied with accurate measurement of glomerular filtration rate (GFR) in pre-clinical studies, clinical practice and clinical trials [1–3]. Inulin [4, 5] and creatinine [6, 7] clearances are accepted reference standards for determining GFR but are expensive and laborious. Colorimetric assays are used to detect Inulin, which is very expensive and also in very short supply and many problems with the assay [8]. 24-h creatinine clearance overestimates GFR in patients with poor renal function and a variety of other glomerular diseases and drugs can make creatinine clearance and serum creatinine a poor markers of GFR estimation [9, 10]. GFR estimated from serum creatinine using prediction equations may result in unpredictable error when the diagnosis of chronic kidney disease is unknown [1–3, 6, 7, 11–23]. Cystatin C, a low molecular weight protein that is produced by nucleated cells at a constant rate and is then filtered by glomeruli and reabsorbed and catabolized by tubular epithelial cells, has been suggested as an alternative for creatinine [24–26]. It seems to be affected by factors other than renal function alone [27–33]. Usually, iothalamate (IOT) clearance [2, 3, 15, 20, 22, 34, 35] are used to measure GFR. Mostly, radioactive compounds are also used to perform IOT clearances [36–38], which have radioactive health and safety issues. That’s why we have used non-radioactive IOT for analysis.

Recently, several high-performance liquid chromatography (HPLC) techniques have been published for the analysis of IOT in serum and urine, but are not without problems [8, 39–46]. Initial methods for sample preparation consisted of several time-consuming extraction and evaporation steps. Also, the sensitivity of some of the previously reported assays is not great, for example, an HPLC–UV method published by Farthing et al. had a linear range for the detection of IOT in plasma from 10 to 50 µg/mL [40]. Later developed procedures reduced sample preparation to a single precipitation step or an ultrafiltration step prior to analysis. LC–MS methods are also used [36, 47] along with capillary electrophoresis [34], both of which are more expensive and require specialized training and expertise to operate.

Herein we report on a method using standard C18 columns, which allow estimation of IOT in a single chromatographic run along with a simple and inexpensive sample preparation method using acetonitrile precipitation as compared to ultrafiltration as done in the past. We don’t require a very expensive instruments to carry out such analysis. Our extraction procedure does not require extensive sample extraction procedures, shows no peak deterioration with high injection volume. The sample preparation method is a much improved version of existing methods [8, 41, 43, 48], notably because the present work describes a more sensitive and simple assay for IOT in a relatively large number of patients, who are given a new formulation of IOT (Conray 43™). Furthermore, IOT concentrations were determined in both, plasma as well as urine samples to calculate GFR, and lastly, we report a fully validated assay according to FDA guidelines.

Our aim was to develop an inexpensive, robust and reliable HPLC method for analysis of IOT in human plasma and urine along with comparing two sample preparation methods (ACN precipitation method compared to ultrafiltration) using two internal standards allopurinol and BHET.

## Experimental

### Materials

Iothalamic acid (P/N USP34500-2) was bought from Promochem, Welwyn Garden City, Herts, UK. Internal Standards Allopurinol (P/N H-9006), Hydroxyethyl-Theophylline (BHET) (P/N H-9006) were purchased from Sigma, Chemical Co, Poole, Dorset, UK, as was Sodium phosphate (Monobasic-ACS reagent grade). HPLC grade acetonitrile, methanol and water were purchased from Hichrom Ltd, Berkshire, UK. Drug free control serum was purchased from Charter House Clinical Research Unit, Slough, UK.

Micro-centrifuge tubes 1.5 mL, Kimble Amber auto-sampler vials, glass inserts, caps and 12 × 75 mm test tubes were all bought from Fisher Scientific Loughborough, UK. Other instruments used were heater block with nitrogen cannula to evaporate samples, platform mixer or vortex to mix samples.

### Apparatus and chromatographic conditions

Hewlett Packard model 1100 high performance liquid chromatography (HPLC) system consisted of HP 1100 series degasser, quaternary pump, auto-injector, column heater and a UV detector. The system was controlled through Chemstation software, version B.04.02 loaded on a HP (Vectra Vm) series computer with a printer. Mobile phase consisted of Methanol: 50 mM Sodium Phosphate (10:90) (unadjusted about PH 4.67). Flow rate was 1.2 mL/min, column used was Synergi-4µ-hydro-RP-80A (250 × 4.6 mm) with a guard column Symmetry Shield RP-18 (15 mm × 4.5 mm, 5 µm) with an ultraviolet Detector set at 254 nm, column temperature was set to 30 °C, injection volume was 10 µL, run time for the run was 15 min. All analyses were performed at room temperature.

### Solution preparation

#### Stock solutions

Preparation of stock iothalamic acid solutions 10 mg/mL. Solutions were prepared in duplicate one for calibration standards and one for quality control. 100 mg of iothalamic acid was weighed out and placed in a 10 mL volumetric flask. Then dissolved in 10 mL methanol and stored in the refrigerator (0–5 °C) (Final conc. = 10 mg/mL). 10 mg of Allopurinol was weighed out and placed in a 10 mL volumetric flask. It was dissolved in 10 mL of methanol and stored in the refrigerator (0–5 °C) (Final conc. = 1.0 mg/mL). β-Hydroxyethyl-Theophylline (BHET), stock internal standard (IS) solution was prepared having a concentration of 1.0 mg/mL. 10 mg of BHET was weighed out and placed in a 10 mL volumetric flask. It was dissolved in a 10 mL of methanol. The solutions were stored in the refrigerator at 0–5 °C (Final conc. = 1.0 mg/mL) and these solution was marked as IS-Stock.

#### Working solutions, calibrants and quality controls

Working internal standard solution was prepared by diluting 1.0 mL of IS Stock solution in a 10 mL volumetric flask and diluted to 10 mL with acetonitrile (final conc. 0.1 mg/mL Allopurinol). Working iothalamic standard solutions were prepared by dissolving 1 mL of stock iothalamic (IOT) solution (conc. = 10 mg/mL) in a 10 mL volumetric and diluted with 9 mL of methanol. Iothalamic acid stock solutions were stored in 15 mL polypropylene screw cap test-tube and stored at 0–5 °C (refrigerated). Calibrants were prepared by serial dilutions and then the volumes were pipetted into clean, labelled 1.5 mL micro-centrifuge tubes, and evaporated to dryness and reconstituted with 100 µL of control plasma or urine to obtain the desired concentrations. Quality control solutions were prepared in the concentration of 1, 2, 20 and 120 µg/mL for plasma and 25, 40, 200 and 1200 for urine. Calibration curve were made in plasma in the range 1–150 µg/mL and in urine in the range 25–1500 µg/mL.

### Phosphate buffer

50 mM monobasic sodium phosphate was prepared by weighing out 6 g of sodium phosphate (Monobasic) and dissolving in 1000 mL of HPLC water, and stored at room temperature.

### Sample preparation and extraction

For plasma samples, working standards and controls were evaporated under a stream of nitrogen and 100 µL of control plasma was added for calibrants and quality control samples preparation, the samples were mixed vigorously for 5–10 s using a platform (vortex) mixer. 50 μL of working internal standard solution of allopurinol was added to each sample, standard and quality control. Next 300 μL of acetonitrile was added to each sample, standard and quality control. All samples were mixed vigorously and centrifuged at 1200×*g* for 10 min at room temperature (18–25 °C). The supernatant were transferred into clean, labelled 12 × 75 mm tubes and evaporated to dryness under a gentle stream of nitrogen. The dried residue was reconstituted with 100 µL of mobile phase and transferred to a glass insert inside a 2 mL standard HPLC auto injector vial. These were vortex mixed and centrifuged for 5 min at 1200×*g* and 10 µL of the supernatant injected onto the HPLC system.

For urine samples, working standards and quality controls were evaporated under a stream of nitrogen and reconstituted in 100 μL of tenfold diluted blank urine. These were mixed vigorously for 5–10 s using a platform (vortex) mixer. This urine solution was then placed in a 1.5 mL micro-centrifuge tube and 50 μL mobile phase containing the internal standard (100 μg/mL Allopurinol) was added to each sample, standard and quality controls. These are mixed vigorously and transferred to a glass insert inside a Kimble vial and 10 μL was injected onto the HPLC system.

### Calculations

Peak areas/heights for each peak were obtained from the computer data capture system. The standard curves were generated by weighted (1/y^2^) linear regression of peak area/height ratios of IOT to allopurinol versus supplemented urine concentrations. Quantitation of unknown samples were estimated by applying the linear regression equation of the standard curve to the unknown sample’s peak area/height ratio.

### Validation

All frozen samples (control and test serum samples) were thawed for approximately half an hour at room temperature, vortexed before processing and transferred to appropriately labelled tubes. The usual analytical run consisted of two control blank matrix samples, calibrators S1-S8, quality control samples (minimum two sets of quality control low -QCL, quality control medium—QCM and quality control high–QCH per run), one set run after blanks and calibrators and then after approximately every 15 test samples. Mobile phase was injected between the analytical control samples and the blanks and also after the calibrators to minimise carryover. Each analytical run was prepared and assayed within a 28-h period.

A quality control was rejected on the standard curve estimations if deviated 15% or more, or in case of limit of quantitation (LOQ), quality controls deviating 20% or more was rejected. Any sample was discarded if a peak of interest had obvious chromatographic interference. A sample, standard, or quality control sample was rejected if the internal standard (peak height/area/injected volume) was one standard deviation from the normal.

### Precision/accuracy and LOQ

The accuracy and precision of the method was determined by assaying 1 mL aliquots of blank human plasma and urine was fortified with four quality control (QC) samples of concentrations 1, 2, 20 and 120 µg/mL for plasma and 25, 40, 200 and 1200 µg/mL for urine. To assess the inter-assay precision and accuracy, samples were analysed on five separate days. To assess the intra-assay precision, these same QC concentrations were analysed during 1 day. Precision and accuracy are reported as percent coefficient of variation and percent accuracy [(observed − expected) × 100/expected concentration], respectively.

The results of QC sample analysis provide the basis for accepting or rejection the run. Variability of the quality control samples was assessed from the validation procedure. For each individual QC, the acceptance criterion was not more than a 15% deviation from the nominal value for accuracy. LOQ was defined as the lowest concentration of an analyte in a sample that was determined with acceptable precision and accuracy under the stated operational conditions of the method. An acceptable range for LOQ was nominal value ±20%.

### Specificity

The responses of interfering peaks at the retention time of the analyte were less than 20% of the response of an LOQ standard. Responses of interfering peaks at the retention time of the internal standard were ≤5% of the response of the concentration of the internal standard to be used in studies which demonstrated the specificity of the validated analytical procedure.

### Linearity

The ratio of UV area response for the peak corresponding to iothalamic acid in the HPLC chromatogram to the UV area response of the internal standard against the nominal concentrations of iothalamic acid represents the linearity. Applying linear regression to the calibration data obtained from seven calibration standards gives the equation of the linear line. A calibration point was rejected as an outlier if the back-calculated concentration for a calibrator (on the basis of the corresponding calibration curve) deviated more than 15% at all concentrations covered by the calibration range except at LOQ where the deviation of 20% was acceptable. A calibration curve was accepted with a minimum of 6 out 8 acceptable calibration levels.

### Recovery

The recovery of IOT from urine and plasma was determined by calculating the ratio of slopes of IOT standard curves against the slope of the same standards prepared in distilled deionized water.

### Stability

Human plasma at concentration of QCL-2, QCM-20 and QCH-120 µg/mL and urine samples at concentrations QCL-40, QCM-200 and QCH-1200 were subjected to 3 freeze and thaw cycles. The results obtained after each freeze and thaw cycle was expressed as a percentage change from the results for QCL-40, QCM-200 and QCH-1200 in the intra-assay run (validation run-1, these samples were prepared fresh and had not experienced any freezing conditions). The test compound was considered to be stable if the percentage change from freshly prepared samples was within ±15% of the nominally spiked level.

### GFR calculations

An effective way of assessing how well the kidneys are working is to calculate the glomerular filtration rate (GFR). GFR is a measurement of how many millilitres (mL) of waste fluid the kidneys can filter from the blood in a minute (measured in mL/min). A healthy pair of kidneys should be able to filter more than 90 mL/min. The result is called the estimated GFR or eGFR.

The IOT clearance was calculated by the formula

$${\text{GFR}} = \frac{{{\text{Urine Concentration}} \times \,{\text{Urine Flow}}}}{\text{Plasma Concentration}}$$

Here glomerular filtration rate was calculated by multiplying urine flow rate with concentration of IOT in urine divided by IOT concentration in plasma. The GFR was recorded in units of volume per time i.e.; mL/min. The following table shows the different stages of renal function [49]. The urine flow was measured using uroflowmetry device, Flow-Med™ 1100 and measuring flow of urine over time.

### Subjects

Twenty healthy young volunteers were recruited for the study. Each volunteer received a 1.5 mL bolus injection of Conray™ 43 containing 645 mg of iothalamate meglumine (Malinkrodt Plc, Ireland) and a 5-mL sterile normal saline flush. To estimate GFR over 150 min period blood and urine samples were collected. Blood and urine samples were first collected after 60 min and then samples were collected every 30 min for three consecutive time interval. All samples were stored at −20 °C until analysed (within 1 month). Kingston University research ethics committee approved the study protocol and the volunteers provided informed written consent to participate in the study. This study was conducted according to the principles of the Declaration of Helsinki [50].

## Results and discussion

### Chromatography

**Fig. 1:**
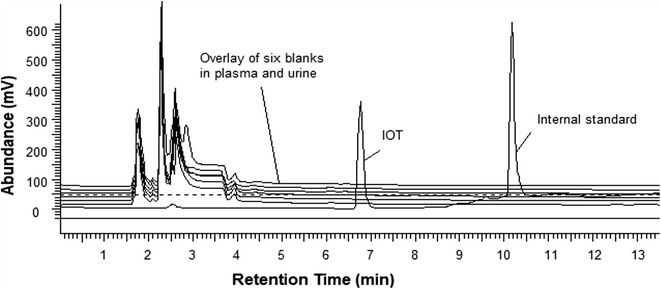
A typical chromatogram is shown for iothalamic acid and internal standard (Allopurinol) in human plasma and urine with blanks overlay to show specificity

A representative chromatogram of IOT and internal standard, are shown in Fig. 1. A representative chromatogram, showing an overlay of blank human serum and urine along with a representative chromatogram of urine and plasma, after injecting IOT in a human subject, is shown in Fig. 1. The chromatographic conditions were adapted from a previously published report [26]. The mobile phase elution of 10% methanol and 90% 50 mM sodium phosphate in water gave maximum separation. Both IOT and the internal standard showed sharp, well-defined peaks at retention times of 6.9 and 10.4 min, respectively.

### Intra-day and inter-day precision and accuracy

The accuracy and precision of the method was determined by assaying 100 μL aliquots of human plasma fortified with 1, 2, 20 and 120 μg/mL of iothalamic acid (representing quality controls: LOQ, QCL, QCM and QCH respectively) and 25, 40, 200 and 1200 concentrations for human urine. These fortified samples were assayed by HPLC–UV. Intra-assay accuracy and precision for iothalamic acid in urine were calculated from results obtained from 6 replicate analyses of quality controls each at 4 concentrations (1, 2, 20 and 120 μg/mL of iothalamic acid in human plasma, and 25, 40, 200 and 1200 concentrations for human urine). Different stanges of renal function are given in Table 1 below.

**Table 1:** Different stages of renal function

Stages	GFR (mL/min)	Kidney function
Stage 1	≥90	Normal
Stage 2	60–89	Slight decrease
Stage 3	30–44	Mild decrease
Stage 4	15–29	Severe decrease
Stage 5	≤15	Renal failure

The intra and  inter assay results show 1 μg/mL to be the limit of quantitation level for iothalamic acid in human plasma and 25 μg/mL for urine, using developed analytical method. These are summarised below in Table 2.

**Table 2:** Summary of assay validation results

Analyte (µg/mL)	QC	Linear range	LOD	R^2^	Intra-day (N = 6)		Inter-day (N = 12)		Recovery
					Precision, % CV	Accuracy %	Precision, % CV	Accuracy %	
Iothalamic acid in plasma	1	1–150	0.5	0.9985	4.56	86.33	13.16	90.08	
	2				9.46	112.5	12.87	102.58	
	20				14.96	100.74	11.52	96.6	96.05
	120				2.66	99.88	2.65	98.99	118.38
Iothalamic acid in urine	25	25–1500	5	0.9992	4.31	97.54	4.45	95.91	
	40				3.96	88.49	3.86	90.91	
	200				2.23	114.96	6.68	108.33	93.14
	1200				1.81	109.8	8.56	101.66	111.74

These values were well within the assay criteria for accuracy of nominal concentration ±15%

### Specificity

Chromatogram showing plasma and urine samples from six subjects without any interference from endogenous compounds in the retention time regions of the internal standard and iothalamic acid.

### Linearity

The plots were linear over the concentration range 1–150 μg/mL IOT in human plasma and the fit statistically tested by slope, intercept and correlation coefficient (r^2^) and linear regression with 1/y*y fit was found to be suitable. For urine the plots were linear over the concentration range 25–1500 μg/mL. None of the intercepts were significantly different from zero.

### Recovery of calibration standards

Determination of recovery of iothalamic acid from human plasma and urine was determined by taking the UV area ratio response of iothalamic acid to internal standard in the extracted sample divided by the area ratio response determined in an un-extracted sample and multiplied by 100 gave the percent recovery.

### Stability of iothalamic acid in plasma to repeated freezing and thawing cycles

The QCL-2 µg/mL samples gave a mean result of 1.97, 1.87 and 2.37 μg/mL (n = 6) with the corresponding percentage change from freshly prepared samples of 12.44, 12.59 and −5.19% for freezing and thawing cycles 1,2 and 3 respectively. The QCM-20 samples gave a mean result of 21.37, 20.73 and 20.20 μg/mL (n = 6) with the corresponding percentage change from freshly prepared samples of −6.08, −2.87 and −0.26% for freezing and thawing cycles 1, 2 and 3 respectively. The QCH-120 samples gave a mean result of 122.12, 121.99 and 121.71 μg/mL (n = 6) with the corresponding percentage change from freshly prepared samples from time t = 0 to −1.89, −1.49 and −1.54% for freezing and thawing cycles 1, 2 and 3 respectively. The data indicates that iothalamic acid was stable in plasma to at least 3 freezing and thawing cycles.

### Stability of iothalamic acid in urine to repeated freeze/thaw cycles

The QCL-40 samples gave a mean result of 37.63, 40.19 and 36.07 μg/mL with the corresponding percentage change from freshly prepared samples of +6.31, −13.54 and +1.88% for freeze and thaw cycles 1, 2 and 3 respectively. The QCM-200 samples gave a mean result of 208.34, 211.68 and 213.08 μg/mL with the corresponding percentage change from freshly prepared samples of −9.38, +7.93 and −7.90% for freeze and thaw cycles 1, 2 and 3 respectively. The QCH-1200 samples gave a mean result of 1144.84, 1192.05 and 1227.22 μg/mL with the corresponding percentage change from freshly prepared samples of −13.11, +9.53 and −7.37% for freeze and thaw cycles 1, 2 and 3 respectively. The data indicates that iothalamic acid was stable in urine to at least 3 freeze and thaw cycles.

### Applications in humans

**Fig. 2:**
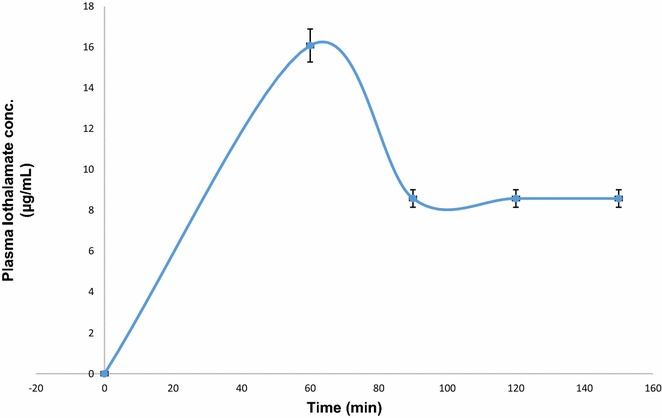
Plasma concentrations of iothalamate in 20 subjects after a single intravenous bolus injection of 1.5-mL Conray™ 43. Plasma iothalamate concentrations ranged between 4.32 and 16.36 μg/mL in the 20 subjects, *error bars* represents standard error of mean (SEM)

Plasma IOT concentration was plotted against time after a single injection (1.5-mL Conray™ 43) as shown in Fig. 2. 0 point on the graph shows the time before the bolus injection was administered. Since the first sample collection and measurement was carried out at 60 min, it is quite likely that the peak concentration might have spiked at a much earlier time point than 60 min. However, the main aim of this part of the study was to measure GFR rates. Figure 2 shows that on the average the IOT reaches its peak plasma concentration in an hours’ time and then it starts to decline. The concentration of IOT in plasma ranged between 4.32 and 16.36 µg/mL in 20 healthy volunteers.

**Fig. 3:**
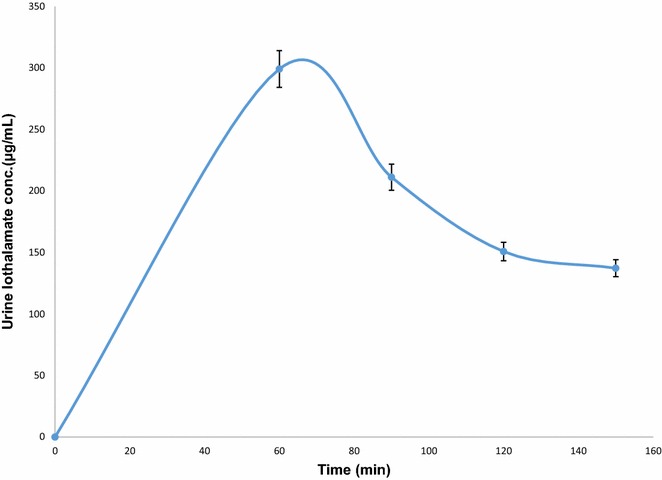
Urine concentrations of iothalamate in 20 subjects after a single intravenous bolus injection of 1.5-mL Conray™ 43. Urine iothalamate concentrations ranged between 76.92 and 311.32 μg/mL in the 20 subjects, here *error bars* represent SEM

Figure 3 shows how the urine IOT concentration changed over time after a single injection (1.5-mL Conray™ 43). The figure also shows how the average concentration of IOT clearance reaches its maximum in an hour time and then it starts to decline. The concentration of IOT in urine ranged between 76.92 and 311.32 µg/mL in 20 healthy volunteers.

**Fig. 4:**
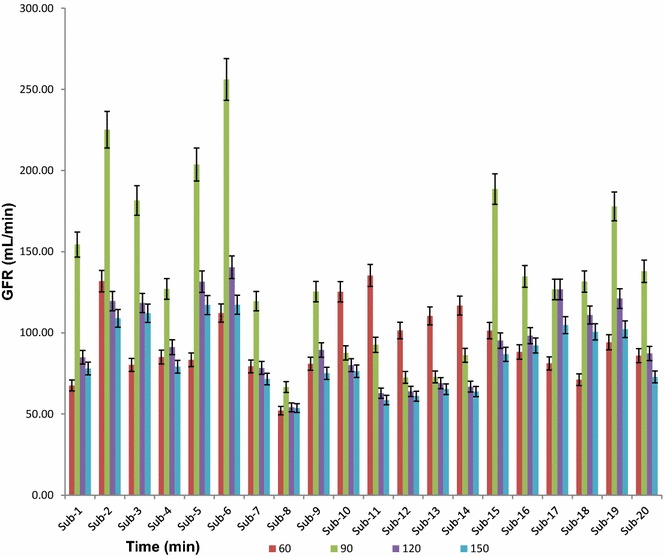
GFR- time profile of 20 volunteers, where blood and urine was collected at 60, 90, 20 and 150 min from subjects after a single intravenous bolus injection of 1.5-mL Conray™ 43, here *error bars* represents SEM

Figure 4 shows the GFR value of 20 volunteers in mL/min against time. GFR was calculated by dividing (concentration of urine excreted × flow of urine) by concentration of plasma for each subject. The 5 columns under each subject represent the five time points of sample collection (0, 60, 90, 120 and 150). Subjects 1, 2, 3, 4, 5, 6, 7, 9, 15, 16, 18, 19 and 20 shows that the glomerular filtration rate was optimum at around 90 min, and subjects 10, 11, 12, 13 and 14 show a high renal clearance at 60 min, while 17 shows maximum GFR from 90 to 120 min. Moreover, subject 8 shows maximum GFR before IOT injection at 0 min.

**Fig. 5:**
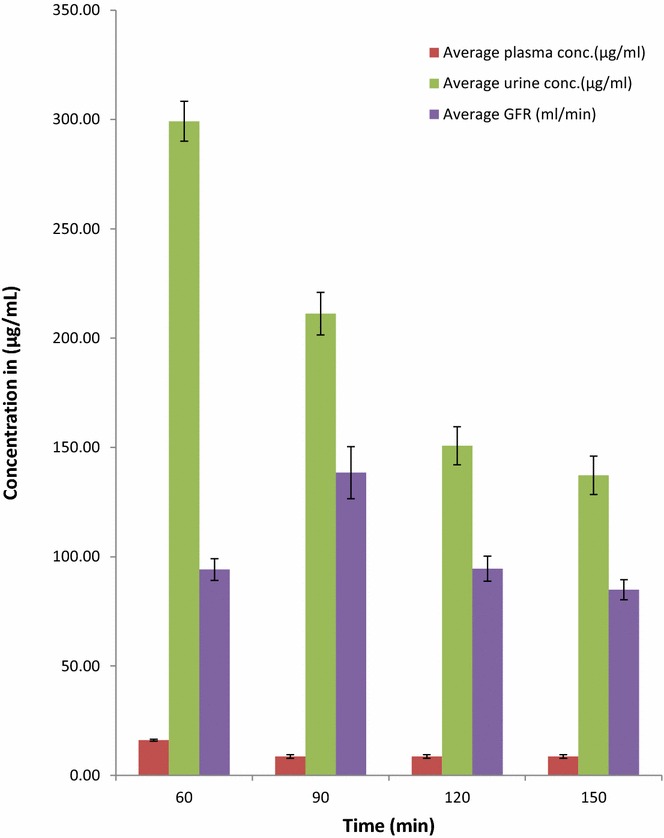
Average plasma, urine and GFR concentration-time profile of 20 volunteers, where blood and urine was collected at 60, 90, 120 and 150 min from subjects after a single intravenous bolus injection of 1.5-mL Conray™ 43, here *error bars* represents SEM

Figure 5 shows a comparative concentration–time profile of 20 volunteers, where blood and urine was collected at 60, 90, 120 and 150 min from subjects after a single intravenous bolus injection of 1.5-mL Conray™ 43. The figure describe how the GFR varies between subjects at different time intervals.

In conclusion, we report on a new, simple and sensitive method for the measurement of IOT, which was then validated and used to estimate GFR. The current method employed a new internal standard, allopurinol (after trying BHET as well) and had the added advantage of only a single step extraction from plasma and urine. The method was further validated in healthy volunteers and showed that GFR was rapidly estimated with minimal blood and urine sampling. GFR determination by IOT clearance is safe in diabetic patients with mild to moderate renal impairment [51]. Furthermore, single plasma sampling 4 h after injection of IOT was safely and accurately undertaken to determine GFR. This enables the technique to be used more frequently in clinical practice as it reduces the cost of GFR determination, and allows the procedure to be undertaken easily in an outpatient setting.

## References

[1] Lamb EJ, Brettell EA, Cockwell P, Dalton N, Deeks JJ, Harris K, Higgins T, Kalra PA, Khunti K, Loud F (2014). The eGFR-C study: accuracy of glomerular filtration rate (GFR) estimation using creatinine and cystatin C and albuminuria for monitoring disease progression in patients with stage 3 chronic kidney disease-prospective longitudinal study in a multiethnic population. BMC Nephrol.

[2] Gaspari F, Ruggenenti P, Porrini E, Motterlini N, Cannata A, Carrara F, Sosa AJ, Cella C, Ferrari S, Stucchi N (2013). The GFR and GFR decline cannot be accurately estimated in type 2 diabetics. Kidney Int.

[3] Francoz C, Nadim MK, Baron A, Prié D, Antoine C, Belghiti J, Valla D, Moreau R, Durand F (2014). Glomerular filtration rate equations for liver-kidney transplantation in cirrhotic patients: validation of current recommendations. Hepatology.

[4] Baccard N, Hoizey G, Frances C, Lamiable D, Trenque T, Millart H (1999). Simultaneous determination of inulin and p-aminohippuric acid (PAH) in human plasma and urine by high-performance liquid chromatography. Analyst.

[5] Maher F, Nolan, N, Elveback L (1971) Comparison of simultaneous clearances of 125-I-labeled sodium lothalamate (Glofil) and of inulin. In: Mayo Clinic proceedings 5115749

[6] Rule AD, Bailey KR, Lieske JC, Peyser PA, Turner ST (2013). Estimating the glomerular filtration rate from serum creatinine is better than from cystatin C for evaluating risk factors associated with chronic kidney disease. Kidney Int.

[7] Levey AS, Coresh J, Greene T, Stevens LA, Zhang YL, Hendriksen S, Kusek JW, Van Lente F (2006). Using standardized serum creatinine values in the modification of diet in renal disease study equation for estimating glomerular filtration rate. Ann Intern Med.

[8] Agarwal R, Vasavada N, Chase SD (2003). Liquid chromatography for iothalamate in biological samples. J Chromatogr B.

[9] Branten AJ, Vervoort G, Wetzels JF (2005). Serum creatinine is a poor marker of GFR in nephrotic syndrome. Nephrol Dial Transplant.

[10] Mussap M, Dalla Vestra M, Fioretto P, Saller A, Varagnolo M, Nosadini R, Plebani M (2002). Cystatin C is a more sensitive marker than creatinine for the estimation of GFR in type 2 diabetic patients. Kidney Int.

[11] Johnson CA, Levey AS, Coresh J, Levin A, Lau J, Eknoyan G (2004). Clinical practice guidelines for chronic kidney disease in adults: part II. Glomerular filtration rate, proteinuria, and other markers. Am Fam Phys.

[12] Al K, YT A, JC J (2005). Definition and classification of chronic kidney disease: a position statement from Kidney Disease: Improving Global Outcomes (KDIGO). Kidney Int.

[13] Lin J, Knight EL, Hogan ML, Singh AK (2003). A comparison of prediction equations for estimating glomerular filtration rate in adults without kidney disease. JASN.

[14] Tsai C-W, Grams ME, Inker LA, Coresh J, Selvin E (2013). Cystatin C-and creatinine-based estimated glomerular filtration rate, vascular disease, and mortality in persons with diabetes in the United States. Diabetes Care.

[15] Rule AD, Gussak HM, Pond GR, Bergstralh EJ, Stegall MD, Cosio FG, Larson TS (2004). Measured and estimated GFR in healthy potential kidney donors. Am J Kidney Dis.

[16] Fontseré N, Salinas I, Bonal J, Bayés B, Riba J, Torres F, Rios J, Sanmartí A, Romero R (2006). Are prediction equations for glomerular filtration rate useful for the long-term monitoring of type 2 diabetic patients?. Nephrol Dial Transplant.

[17] Padmasree D, Kumar MA, Ukey Ujwala U (2013). Glomerular filtration rate in healthy indian adults by the modification of diet in renal disease equation. IJRTSAT.

[18] Valente MA, Hillege HL, Navis G, Voors AA, Dunselman PH, van Veldhuisen DJ, Damman K (2013). The chronic kidney disease epidemiology collaboration equation outperforms the modification of diet in renal disease equation for estimating glomerular filtration rate in chronic systolic heart failure. Eur J Heart Fail.

[19] Kilbride HS, Stevens PE, Eaglestone G, Knight S, Carter JL, Delaney MP, Farmer CK, Irving J, O’Riordan SE, Dalton RN (2013). Accuracy of the MDRD (Modification of Diet in Renal Disease) study and CKD-EPI (CKD Epidemiology Collaboration) equations for estimation of GFR in the elderly. Am J Kidney Dis.

[20] Blake GM, Sibley-Allen C, Hilton R, Burnapp L, Moghul MR, Goldsmith D (2013). Glomerular filtration rate in prospective living kidney donors. Int Urol Nephrol.

[21] Chin P, Florkowski C, Begg E (2013). The performances of the Cockcroft-Gault, modification of diet in renal disease study and chronic kidney disease epidemiology collaboration equations in predicting gentamicin clearance. Ann Clin Biochem.

[22] Bragadottir G, Redfors B, Ricksten S-E (2013). Assessing glomerular filtration rate (GFR) in critically ill patients with acute kidney injury-true GFR versus urinary creatinine clearance and estimating equations. Crit Care.

[23] Willems JM, Vlasveld T, den Elzen WP, Westendorp RG, Rabelink TJ, de Craen AJ, Blauw GJ (2013). Performance of Cockcroft-Gault, MDRD, and CKD-EPI in estimating prevalence of renal function and predicting survival in the oldest old. BMC Geriatr.

[24] Whelly S, Serobian G, Borchardt C, Powell J, Johnson S, Hakansson K, Lindstrom V, Abrahamson M, Grubb A, Cornwall GA (2014). Fertility defects in mice expressing the L68Q variant of human cystatin C: a role for amyloid in male infertility. J Biol Chem.

[25] Abrahamson M, Olafsson I, Palsdottir A, Ulvsback M, Lundwall A, Jensson O, Grubb A (1990). Structure and expression of the human cystatin C gene. Biochem J.

[26] Dönmez O, Korkmaz HA, Yildiz N, Ediz B (2015). Comparison of serum cystatin C and creatinine levels in determining glomerular filtration rate in children with stage I to III chronic renal disease. Ren Fail.

[27] Vega A, de Vinuesa SG, Goicoechea M, Verdalles Ú, Martínez-Pueyo ML, Chacón A, Quiroga B, Luño J (2014). Evaluation of methods based on creatinine and cystatin C to estimate glomerular filtration rate in chronic kidney disease. Int Urol Nephrol.

[28] Nasseri-Moghaddam S, Tofangchiha S, Ganji M (2013). Serum cystatin-C is not superior to serum creatinine in predicting glomerular filtration rate in cirrhotic patients. MEJDD.

[29] Shima T, Khatun A, Yeasmin F, Ferdousi S, Kirtania K, Sultana N (2013). Cystatin C: a better predictor of kidney function in diabetic patients. BSMB.

[30] Nehus EJ, Laskin BL, Kathman TI, Bissler JJ (2013). Performance of cystatin C-based equations in a pediatric cohort at high risk of kidney injury. Pediatr Nephrol.

[31] Ristiniemi N, Savage C, Bruun L, Pettersson K, Lilja H, Christensson A (2012). Evaluation of a new immunoassay for cystatin C, based on a double monoclonal principle, in men with normal and impaired renal function. Nephrol Dial Transplant.

[32] Bansal N, Vittinghoff E, Peralta CA, Shlipak MG, Grubbs V, Jacobs DR, Siscovick D, Steffes M, Carr JJ, Bibbins-Domingo K (2013). Estimated kidney function based on serum cystatin C and risk of subsequent coronary artery calcium in young and middle-aged adults with preserved kidney function: results from the CARDIA study. Am J Epidemiol.

[33] Isakova T, Craven TE, Scialla JJ, Nickolas TL, Schnall A, Barzilay J, Schwartz AV (2015). Change in estimated glomerular filtration rate and fracture risk in the action to control cardiovascular risk in diabetes trial. Bone.

[34] Wilson DM, Bergert JH, Larson TS, Liedtke RR (1997). GFR determined by nonradiolabeled iothalamate using capillary electrophoresis. Am J Kidney Dis.

[35] Barbour GL, Crumb CK, Boyd CM, Reeves RD, Rastogi SP, Patterson RM (1976). Comparison of inulin, iothalamate, and 99mTc-DTPA for measurement of glomerular filtration rate. J Nucl Med.

[36] Rhea JM, Ritchie JC, Molinaro RJ (2013). Development of a liquid chromatography tandem mass spectrometry method for iothalamate measurement to assess renal function for potential kidney donation. Clin Chim Act.

[37] Smith T, Veall N, Altman D (1981). Dosimetry of renal radiopharmaceuticals: the importance of bladder radioactivity and a simple aid for its estimation. Br J Radiol.

[38] Israelit A, Long D, White M, Hull A (1973). Measurement of glomerular filtration rate utilizing a single subcutaneous injection of 125I-iothalamate. Kidney Int.

[39] Hagan AS, Jones DR, Agarwal R (2013). Use of dried plasma spots for the quantification of iothalamate in clinical studies. Clin J Am Soc Nephrol.

[40] Farthing D, Sica DA, Fakhry I, Larus T, Ghosh S, Farthing C, Vranian M, Gehr T (2005). Simple HPLC–UV method for determination of iohexol, iothalamate, p-aminohippuric acid and n-acetyl-p-aminohippuric acid in human plasma and urine with ERPF, GFR and ERPF/GFR ratio determination using colorimetric analysis. J Chromatogr B.

[41] Kos T, Moser P, Yilmatz N, Mayer G, Pacher R (2000). High-performance liquid chromatographic determination of p-aminohippuric acid and iothalamate in human serum and urine: comparison of two sample preparation methods. J Chromatogr B Biomed Sci Appl.

[42] Agarwal R (1998). Chromatographic estimation of iothalamate and p-aminohippuric acid to measure glomerular filtration rate and effective renal plasma flow in humans. J Chromatogr B Biomed Sci Appl.

[43] Bell RR, Bombardt PA, DuCharme DW, Kolaja GJ, Packwood WH, Bothwell BE, Satoh PS (1994). A non-radioactive iothalamate and p-aminohippuric acid high-performance liquid chromatographic method for simultaneously measuring glomerular filtration rate and renal blood flow in the rat. Biomed Chromatogr.

[44] Seneviratne AK, Jayewardene AL, Gambertoglio JG (1994). Paired ion reversed-phase HPLC assay for the determination of iothalamic acid and para aminohippuric acid in urine. J Pharm Biomed Anal.

[45] Gaspari F, Mainardi L, Ruggenenti P, Remuzzi G (1991). High-performance liquid chromatographic determination of iothalamic acid in human plasma and urine. J Chromatogr B Biomed Sci Appl.

[46] Reidenberg M, Lorenzo B, Drayer D, Kluger J, Nestor T, Regnier J, Kowal B, Bekersky I (1987). A nonradioactive iothalamate method for measuring glomerular filtration rate and its use to study the renal handling of cibenzoline. Ther Drug Monit.

[47] Seegmiller JC, Burns BE, Fauq AH, Mukhtar N, Lieske JC, Larson TS (2010). Iothalamate quantification by tandem mass spectrometry to measure glomerular filtration rate. Clin Chem.

[48] Prueksaritanont T, Chen M-L, Chiou WL (1984). Simple and micro high-performance liquid chromatographic method for simultaneous determination of p-aminohippuric acid and iothalamate in biological fluids. J Chromatogr B Biomed Sci Appl.

[49] NHS. Kidney disease, chronic—diagnosis; NHS choices your health, your choices 2012. http://www.nhs.uk/Conditions/Kidney-disease-chronic/Pages/Diagnosis.aspx

[50] World MAGA (2004). World Medical Association Declaration of Helsinki: ethical principles for medical research involving human subjects. J Int Bioethique.

[51] Group, DER (2011). Intensive diabetes therapy and glomerular filtration rate in type 1 diabetes. N Engl J Med.

